# Microbiological Isolates and Antibiotic Susceptibilities in Cases of Posttraumatic Endophthalmitis: A 15-Year Review

**DOI:** 10.1155/2020/5053923

**Published:** 2020-04-29

**Authors:** Chunhong Liu, Jian Ji, Zhujian Wang, Huiwen Chen, Wenjun Cao, Xinghuai Sun

**Affiliations:** ^1^Department of Clinical Laboratory, Eye and ENT Hospital, Shanghai Medical College, Fudan University, Shanghai, China; ^2^Department of Ophthalmology and Visual Science, Eye and ENT Hospital, Shanghai Medical College, Fudan University, Shanghai, China

## Abstract

**Purpose:**

The objective of this study was to evaluate the microbiological spectrum and antibiotic susceptibilities of isolates in posttraumatic endophthalmitis over a 15-year period.

**Methods:**

A retrospective study of 3,163 posttraumatic endophthalmitis cases was conducted between July 2004 and July 2019. The outcome measures included the microbiological spectrum and antibiotic susceptibilities. Chi-squared tests were conducted to detect trends in changes in antibiotic sensitivity over the 15-year period. *P* values of <0.05 were considered statistically significant.

**Results:**

Of the 3,163 cases of posttraumatic endophthalmitis, 1,003 culture-positive isolates were identified. Among these, there were 848 (84.5%) Gram-positive isolates, 109 (10.9%) Gram-negative isolates, and 46 (4.6%) fungal isolates. The most common isolates were Staphylococcal species. There was a significant increase in the percentage of fungal isolates over the 15-year period (*P*=0.02). Gram-positive organisms showed the greatest level of susceptibility to vancomycin (99.6%). The susceptibilities of the 109 Gram-negative isolated organisms were as follows: levofloxacin (95.8%), meropenem (95.7%), ciprofloxacin (93.5%), tobramycin (90.8%), imipenem (88.9%), trimethoprim-sulfamethoxazole (TMP-SMX) (87.7%), ertapenem (80%), and ceftazidime (79.1%). The susceptibility of Gram-positive organisms to several antibiotics, including levofloxacin (*P*=0.004), ciprofloxacin (*P* < 0.001), and chloramphenicol (*P*=0.001) decreased over time, whereas the susceptibility to TMP-SMX increased over time (*P* < 0.001). The susceptibility of Gram-negative bacilli to ceftazidime decreased over time (*P*=0.03).

**Conclusions:**

Over the 15-year study period, most isolates were Gram-positive cocci, especially coagulase-negative staphylococci (CNS). Vancomycin seemed to be the most effective antibiotic for Gram-positive bacteria. Gram-negative bacteria appeared to be most susceptible to fluoroquinolones. A number of antibiotics showed an increasing trend of microbial resistance.

## 1. Introduction

Endophthalmitis is a devastating clinical condition that can lead to severe visual loss. [1–4] Previous research reported that the incidence rate of infectious endophthalmitis following intraocular foreign body (IOFB) injuries ranged from 6.9–30% [5]. IOFB injuries were reported in 43% of posttraumatic endophthalmitis cases [6]. A number of studies showed that the specific characteristics of the IOFB, microorganisms, and time between the injury and treatment were associated with an increased risk of endophthalmitis following penetrating trauma [7–9]. An understanding of the spectrum of pathogens and their antibiotic susceptibilities is essential to guide first-line empirical treatment. Information on the microbial spectra and antibiotic susceptibilities of microbes involved in endophthalmitis following IOFB injuries is limited, with no studies on this issue in East China.

The purpose of this study was to investigate the spectrum of pathogens in culture-proven endophthalmitis and related antibiotic susceptibilities at a tertiary hospital in Shanghai.

## 2. Materials and Methods

This was a retrospective review of 3,163 patients admitted to the Eye, Ear, Nose, and Throat (ENT) Hospital, Shanghai Medical College from 1 July 2004 to 31 July 2019. The study was performed in compliance with the principles of the Declaration of Helsinki and was approved by the ethics committee of the Eye and ENT Hospital (no. 2015011). Informed consent was not required as the data were obtained from patients' clinical records in the medical database of the Eye and ENT Hospital.

The ocular examination included the collection of IOFB, aqueous, and vitreous samples. The samples were inoculated onto blood agar, chocolate agar, and brain heart infusion broth and incubated at 37°C. For fungal cultures, the specimens were inoculated on Sabouraud's dextrose agar and incubated at 25°C. Bacterial identification was performed using a MicroScan AutoScan system (Dade MicroScan Inc., Sacramento, CA, USA). Antibiotic susceptibility testing was performed using a MicroScan AutoScan system (Dade MicroScan Inc., Sacramento, CA, USA) or the *E* test (bioMérieux, France). Antibiotic susceptibility was determined according to the guidelines of the Clinical and Laboratory Standards Institute.

All analyses were performed using SPSS, version 21.0 for Windows (SPSS Inc., Chicago, IL, USA). In the statistical analyses, Chi-squared tests were used to detect trends. *P* values <0.05 were considered significant.

## 3. Results

Over the 15-year study period, 1,003 culture-positive isolates were identified among the 3,163 cases of IOFB injuries. The mean age of the patients was 37 ± 15 y, and 90.6% of the patients were males.

### 3.1. Microbiological Spectrum

In terms of the microbiological spectrum, there were 848 (84.5%) Gram-positive isolates, 109 (10.9%) Gram-negative isolates, and 46 (4.6%) fungal isolates. The most common organisms were Staphylococcal species, with *Staphylococcus epidermidis* in 303 cases, other CNS in 259 cases, and *Staphylococcus aureus* in 113 cases.  Table 1 presents a detailed overview of the microbial isolates.

### 3.2. Changes in the Microbiological Spectrum

**Figure 1:**
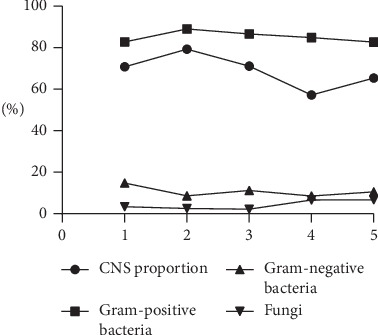
Time trends of different isolated organisms from 2004 to 2019. 1, July 2004 to June 2007; 2, July 2007 to June 2010; 3, July 2010 to June 2013; 4, July 2013 to June 2016; 5, July 2016 to July 2019.

As shown in Figure 1, the percentage of fungal isolates increased significantly occurred over the 15-year period (*P*=0.02). Trends for other isolates were not statistically significant. Trends in the percentage of Gram-positive bacteria (*P*=0.797) and Gram-negative bacteria (*P*=0.586) and the proportion of CNS among Gram-positive bacteria (*P*=0.203) did not reach statistical significance.

### 3.3. Antibiotic Susceptibilities of the Cultured Gram-Positive Organisms

Table 2 provides information on the antibiotic susceptibilities of the Gram-positive organisms identified. The majority of Gram-positive organisms showed susceptibility to vancomycin (99.6%), followed by moxifloxacin (90.9%), levofloxacin (82.3%), ofloxacin (78.1%), chloramphenicol (78.0%), and ciprofloxacin (64.7%). Staphylococcal species were highly susceptible to vancomycin (100%).

### 3.4. Antibiotic Susceptibilities of the Cultured Gram-Negative Organisms

The antibiotic susceptibilities of the 109 Gram-negative isolates were as follows: levofloxacin (95.8%), meropenem (95.7%), gentamicin (95.7%), amikacin (94.6%), ciprofloxacin (93.5%), tobramycin (90.8%), imipenem (88.9%), TMP-SMX (87.7%), ertapenem (80%), and ceftazidime (79.1%) (Table 3).

### 3.5. Time Trends in Bacterial Susceptibilities to Antibiotics

**Figure 2:**
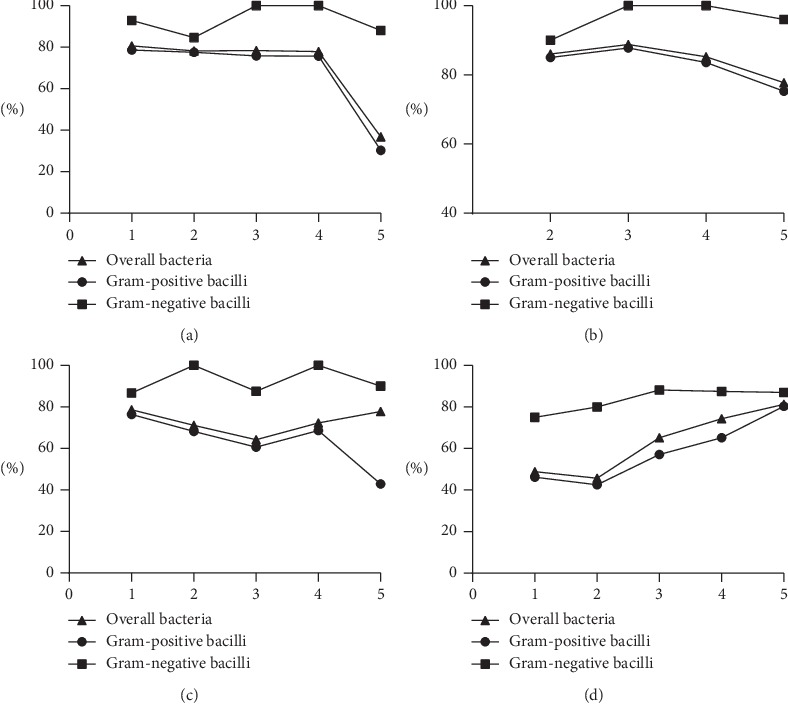
Time trends of bacterial susceptibilities. 1, July 2004 to June 2007; 2, July 2007 to June 2010; 3, July 2010 to June 2013; 4, July 2013 to June 2016; 5, July 2016 to July 2019. Susceptibility testing was unavailable for levofloxacin from July 2004 to July 2007. (a) Ciprofloxacin. (b) Levofloxacin. (c) Tobramycin. (d) TMP-SMX.

Chi-squared tests were performed to examine trends in susceptibilities. The results are shown in Figure 2. The susceptibility of Gram-positive organisms to several antibiotics, including levofloxacin (*P*=0.004), ciprofloxacin (*P* < 0.001), and chloramphenicol (*P*=0.001) decreased over time, whereas the susceptibility to TMP-SMX (*P* < 0.001) increased over time. The susceptibility of Gram-negative bacilli to ceftazidime (*P*=0.03) decreased over time. Trends in susceptibilities for other organisms were not statistically significant (Table 4).

## 4. Discussion

Many studies have described series of posttraumatic endophthalmitis and the distribution of isolates worldwide [1, 6, 10, 11]. These studies showed that the susceptibilities of microbiological isolates and infectious agents to particular antibiotics varied over time and differed according to regional variability, population, and ethnicity [12–16]. The findings of these studies point to the importance of regular periodic reviews of local susceptibilities to ensure that the most appropriate antibiotics are used to treat infections.

Previous research reported positive cultures in 6.9–43% of posttraumatic endophthalmitis cases [3]. Cultures were positive in 31.7% of eyes in the present series. In terms of the distribution of these isolates, the microbiological spectrum in the present study was generally similar to that found in other reports. [17] CNS were the most common causative pathogen in this study, which agreed with the findings of previous studies [6, 16, 18, 19]. In contrast, CNS were involved in 56.0% of endophthalmitis cases in our study versus 23.1% in a study conducted in France [20] (Table 1). A previous study reported a high incidence of endophthalmitis caused by Staphylococcal species following open-globe injuries [11]. In these cases, endophthalmitis was likely the result of normal skin flora and contamination of open wounds [18]. Studies also reported that *Pseudomonas* and *Clostridium* species caused fulminant endophthalmitis [21, 22]. Fungal endophthalmitis is less common than bacterial endophthalmitis, which should be suspected in cases of wood or soil contamination in mild and moist climates. The incidence of fungal isolates in our study was lower than that reported in a previous study (4.6% vs. 16.8%) [13].

In terms of antibiotic sensitivities and empirical antibiotics, our study confirmed that Gram-positive isolates were generally susceptible to vancomycin (Table 2), which was consistent with the findings of previous reports [16, 23, 24]. The high susceptibility rate of Gram-positive bacteria to vancomycin (99.6%) supports its continued use as empirical therapy. In previous studies, the sensitivity of Gram-negative isolates to ceftazidime differed according to regional variability, with figures of 91.5%, 80.0%, and 77.2% reported [10, 23, 25]. In our study, 79.1% of Gram-negative organisms were susceptible to ceftazidime. In addition, the susceptibility of Gram-negative organisms to ceftazidime decreased over time (*P*=0.03). These findings raise questions around the potential need for changes in empirical therapy, as well as whether intravitreal fluoroquinolone can be a substitute for ceftazidime. Changes in empirical therapy would need to be guided by local microbiological susceptibility patterns. Our series confirmed that the susceptibility of Gram-negative isolates to fluoroquinolones (levofloxacin, 95.8%; ciprofloxacin, 93.5%) was greater than their susceptibility to ceftazidime (79.1%). Previous studies demonstrated that ciprofloxacin penetrated the blood-ocular barrier, pointing to its use as an intraocular drug [26, 27]. Ocular toxicity appears to be dose-dependent and results from class-effects and specific fluoroquinolone structures, which has been investigated only in animal models [28, 29]. However, the reports regarding the safety of intracameral injection of moxifloxacin has increased [30–34]. Moxifloxacin provides superior coverage against Gram-positive organisms, and it also maintains excellent coverage for Gram-negative organisms [35].

Topical fluoroquinolones, which offer broad-spectrum antimicrobial coverage and good ocular penetration, are increasingly used. A recent study reported resistance of endophthalmitis and other ophthalmic isolates to fluoroquinolones [36]. In the present study, levofloxacin and ciprofloxacin exhibited a trend toward increased resistance to Gram-positive bacteria (*P*=0.004 and *P* < 0.001, respectively). Increased use of topical fluoroquinolones may lead to resistance in bacterial flora and have a detrimental effect on eye health. Such resistance could partially explain the emergence of fluoroquinolone resistance in ocular microbiology [37, 38]. In the present study, the susceptibility rates of Gram-negative microorganisms to fluoroquinolones were higher than those of Gram-positive microorganisms, which paralleled the findings of previous research [23].

## 5. Conclusion

The present study has some limitations. It was a retrospective, laboratory study that lacked clinical data. We had access only to microbiological records. In addition, due to technical limitations, anaerobic cultures were not performed. Finally, we could not confirm a correlation between the in vitro susceptibility of microbiological isolates and clinical outcomes.

In conclusion, in this 15-year study, Gram-positive cocci, especially CNS, were the most common organisms isolated in posttraumatic endophthalmitis. There was a significant increase in the percentage of fungal isolates over time. Vancomycin seemed to be the most effective antibiotic for Gram-positive bacteria. Gram-negative bacteria were most susceptible to fluoroquinolones. The susceptibility of the isolates to several antibiotics decreased over time. Antibiotic resistance remains a challenge. Continued surveillance of microbiological isolates provides critical information to guide selection of the most appropriate antibiotics used for empirical management of endophthalmitis.

## Figures and Tables

**Table 1:** Microbiological spectrum of isolated organisms from 2004 to 2019.

Organisms	*N*	Total (%)
Gram positive	848	84.5
*Staphylococcus epidermidis*	303	30.2
*Staphylococcus aureus*	113	11.3
Other *CNS*	259	25.8
*Streptococcus* species & amp	36	3.6
*Corynebacterium* species	27	2.7
*Bacillus* specie*s*^	97	9.7
Other Gram-positive bacteria	13	1.3
Gram negative	109	10.9
*Enterobacteriaceae*^*∗*^	51	5.1
Gram-negative ^*∗∗*^	23	2.3
Gram-negative bacteria #	35	3.5
Fungi	46	4.6
Total	1003	100

& refres to *alpha hemolytic streptococcus* and *beta hemolytic streptococcus*^ *Bacillus* cereus and *Bacillus* subtilis ^*∗*^*K*. *pneumoniae*, *E. coli*, *Serratia* sp., *Enterobacter* agglomerans, *Proteus* sp., and *Enterobacter* sp. ^*∗∗*^*Acinetobacter* sp., *Haemophilus* sp., *Moraxella* sp., and *Neisseria* sp. #*Flavobacterium breve*, *Pseudomonas maltophilia*, *Vibrio flurialis*, *Flavobacterium indologenes*, *Pseudomonas* stutzeri, *Pseudomonas* oryzihabitans, and *Pseudomonas aeruginosa*

**Table 2:** Antibiotic susceptibilities of Gram-positive organisms from 2004 to 2019.

Antibiotic	Number of susceptible/total number tested	Percent susceptible
Vancomycin	775/778	99.6
Penicillins/beta lactams		
Penicillin G	256/735	34.8
Methicillin/oxacillin	268/680	39.4
Fluoroquinolones		
Ciprofloxacin	493/763	64.7
Ofloxacin	364/466	78.1
Levofloxacin	469/570	82.3
Moxifloxacin	140/154	90.9
Others		
Erythromycin	243/740	32.8
Chloramphenicol	478/613	78.0
TMP-SMX	248/411	60.3
Tobramycin	255/381	66.9

**Table 3:** Antibiotic susceptibilities of Gram-negative organisms from 2004 to 2019.

Antibiotic	Number of susceptible/total number tested	Percent susceptible
Penicillins/beta lactams		
Ampicillin	19/58	32.8
Cephems		
Cefuroxime	26/36	72.2
Ceftriaxone	54/79	68.4
Ceftazidime	72/91	79.1
Aminoglycosides		
Gentamicin	89/93	95.7
Tobramycin	69/76	90.8
Amikacin	87/92	94.6
Fluoroquinolones		
Ciprofloxacin	86/92	93.5
Levofloxacin	68/71	95.8
Others		
Imipenem	64/72	88.9
Meropenem	45/47	95.7
Ertapenem	24/30	80.0
TMP-SMX	64/73	87.7

**Table 4:** Antibiotic susceptibilities trends for organisms from 2004 to 2019.

*P* value^*∗*^	Overall bacteria	Gram-positive bacteria	Gram-negative bacteria
Ciprofloxacin	<0.001	<0.001	0.943
Levofloxacin	0.005	0.004	0.726
Tobramycin	0.629	0.173	0.767
TMP-SMX	<0.001	<0.001	0.416
Chloramphenicol	/	0.001	/
Ceftazidime	/	/	0.003

^*∗*^ *χ*2 test for trend. Underlined values mean statistically significant at *P* < 0.05.

## Data Availability

The data sets used and/or analyzed during the current study are available from the corresponding author on reasonable request.
